# Effect of Statin Potency on Rapid Coronary Intimal Thickening and Rejection in Heart Transplant Recipients

**DOI:** 10.31486/toj.25.0053

**Published:** 2025

**Authors:** Alyssa Stutes, Steven Quoc Thai, Brooke Baetz, Cruz Velasco-Gonzalez, Selim Krim

**Affiliations:** ^1^ Department of Pharmacy, Ochsner Clinic Foundation, New Orleans, LA; ^2^ Ochsner Center for Outcomes Research, Ochsner Clinic Foundation, New Orleans, LA; ^3^ Section of Cardiomyopathy and Heart Transplantation, John Ochsner Heart & Vascular Institute, Ochsner Clinic Foundation, New Orleans, LA; ^4^ The University of Queensland School of Medicine, Ochsner Clinical School, New Orleans, LA

**Keywords:** *Allografts*, *diagnostic imaging*, *graft rejection*, *heart diseases*, *heart transplantation*, *hydroxymethylglutaryl-CoA reductase inhibitors*, *immunologic factors*, *immunosuppressive agents*, *ultrasonography–interventional*, *vascular diseases*

## Abstract

**Background:**

Statins help prevent cardiac allograft vasculopathy (CAV) and rejection in heart transplant recipients. Whether these adverse outcomes can be further attenuated with higher potency statins is unknown.

**Methods:**

In this single-center, retrospective study, we compared outcomes of heart transplant patients who received either a higher dose statin (HDS) or a lower dose statin (LDS) at discharge after transplant. Exclusion criteria were age <18 years old, not prescribed a statin, and any of the following within 1 year: death, loss to follow-up, or incomplete data to determine the primary outcome. The primary outcome was CAV at 1 year, defined as International Society for Heart and Lung Transplantation CAV grade ≥1 on angiography or intravascular ultrasound demonstrating rapid coronary intimal thickening (0.5-mm increase in coronary maximal intimal thickness at 1 year). Secondary outcomes were biopsy-proven acute rejection, treated rejection, hemodynamically unstable rejection, and adverse effects.

**Results:**

The study population consisted of 81 patients in the HDS group and 103 patients in the LDS group. The incidence of CAV was not different in the HDS vs LDS group (32.1% vs 31.1%, respectively; *P*=0.881) despite less biopsy-proven acute rejection (2.5% vs 12.6%, respectively; *P*=0.013) and less treated rejection (2.5% vs 17.5%, respectively; *P*=0.001) in the HDS group. All other secondary outcomes were similar between groups.

**Conclusion:**

Increasing statin intensity in heart transplant patients appears to be safe and may reduce rejection but did not attenuate CAV at 1 year in our population.

## INTRODUCTION

Heart transplant is a definitive treatment for patients with refractory heart failure. In 2023, >5,000 adult heart transplants were performed worldwide.^1^ Median survival has increased to 12.5 years, but the predominant cause of long-term mortality after heart transplant continues to be graft failure, likely from cardiac allograft vasculopathy (CAV).^2^

CAV, a distinct atherosclerosis of the transplanted heart, can lead to allograft dysfunction. Nonimmune and immune factors contribute to inflammatory cell infiltration, smooth muscle proliferation, and lipid deposition within vessels. Compared to native coronary disease, vessel lesions in CAV are circumferential, diffuse, and develop rapidly.^3^ After transplant, CAV occurs in approximately 25% of patients in 5 years and approximately 50% of patients in 10 years.^4^ Nearly 10% of deaths are attributed to CAV after 5 years, but this figure does not include death from graft failure, which may represent undetected CAV.^2^ Treating CAV is challenging, so prevention is paramount.

Meta-analyses of randomized studies have shown that statins decrease mortality when given after transplant, likely by reducing CAV and rejection.^5,6^ However, whether higher statin potency provides an additional benefit is unclear. Two recent (2021 and 2020) observational studies did not detect less CAV with the use of higher potency statins.^7,8^ In these studies, however, CAV was detected by methods such as coronary angiography that may not detect early CAV when vessel lumens can still be patent. Intravascular ultrasound is more sensitive than coronary angiography; by providing a cross-sectional view of the coronary artery, intravascular ultrasound can detect intimal thickening during earlier stages of CAV.^9,10^

Our study explored if higher statin potencies reduced the incidence of CAV at 1 year, as detected by rapid coronary intimal thickening (0.5-mm increase in coronary maximal intimal thickness at 1 year) on intravascular ultrasound or angiography. We also examined the effect of higher potency statins on rejection, as well as adverse effects.

## METHODS

After receiving approval from the Ochsner Clinic Foundation Institutional Review Board, we conducted a single-center, retrospective study of patients who received a first heart transplant from January 2012 through June 2023. Exclusion criteria were age <18 years old, not on a statin at discharge after transplant, death or loss to follow up within 1 year, and missing intravascular ultrasound and/or angiogram data needed to determine the primary outcome. We compared outcomes between patients who received either a higher dose statin (HDS) or a lower dose statin (LDS) at discharge after transplant. HDS was defined as a statin and dose combination expected to reduce low density lipoprotein by ≥37% (atorvastatin ≥20 mg/day, pravastatin ≥80 mg/day, or rosuvastatin ≥5 mg/day).^11^ LDS was defined as a statin and dose combination not meeting HDS criteria.

All outcomes were assessed at 1 year after transplant. The primary outcome was defined as either International Society for Heart and Lung Transplantation (ISHLT) CAV grade ≥1 on angiography or intravascular ultrasound demonstrating a ≥0.5-mm increase in maximal intimal thickness at 1 year compared to baseline in ≥1 matched vessel(s).^9,10,12^ Secondary outcomes were biopsy-proven acute rejection, treated rejection, and hemodynamically unstable rejection. Biopsy-proven acute rejection was defined as ISHLT cellular rejection ≥2R or pathologic antibody-mediated rejection grade ≥2.^13,14^ Patients were considered treated for rejection if they received any of the following: augmented steroid therapy, anti-thymocyte globulin, rituximab, bortezomib, plasma exchange, or intravenous immunoglobulin. Hemodynamically unstable rejection was defined as treated rejection with an acute decrease in left ventricular ejection fraction (<45% or >25% from baseline) or the need for inotropes or mechanical circulatory support.

We also determined the incidence of the following selected statin adverse events: (1) liver dysfunction, defined as aspartate transaminase or alanine transaminase >3 times the upper limit of normal; (2) myalgia, based on documentation of symptoms; and (3) rhabdomyolysis, defined as creatinine phosphokinase >10 times the upper limit of normal.

Statins were routinely given within 2 weeks of transplant. Before August 2018, institutional guidelines suggested pravastatin 40 mg/day, but from August 2018, the guidelines changed to recommending atorvastatin 20 mg/day. Patients with any 3 of the following characteristics were designated as high risk for rejection and given anti-thymocyte globulin induction: Black race, previous pregnancy, age <40 years, calculated panel reactive antibody >0%, or >4/6 human leukocyte antigen mismatches.^15-17^ Routine immunosuppression included tacrolimus, mycophenolate, and steroids. Coronary angiogram and intravascular ultrasound were performed at 6 weeks (baseline) and 1 year. Cardiac biopsies were performed weekly in month 1, biweekly in month 2, once in month 3, and as needed thereafter with guidance from gene expression profiling or suspicion of rejection. Cellular rejection was treated per ISHLT guidelines, and antibody-mediated rejection was treated per physician discretion.^12^

Sixty-two patients were required in each group to achieve 80% power, assuming a CAV incidence of 10% in the HDS group vs 30% in the LDS group. Categorical data were analyzed with the chi-square test and expressed as frequencies and percentages. Normally distributed data were analyzed with the *t* test and reported as means with standard deviations. Nonnormally distributed data were analyzed with the Mann-Whitney *U* test and reported as medians with interquartile ranges. Multivariable logistic regression analysis was performed to assess the association between statin intensity and CAV at 1 year when controlling for anti-thymocyte globulin induction, treated cytomegalovirus viremia, and calculated panel reactive antibody ≥25%. Two-tailed *P* values were considered significant if α <0.05. SAS version 9.4 (SAS Institute Inc) was used to conduct all analyses.

## RESULTS

During the study period, 281 patients received a heart transplant (Figure). After exclusions, 184 patients were analyzed: 81 patients in the HDS group and 103 patients in the LDS group. Most patients were male and White, with a median age of 50 years (Table 1). Baseline characteristics were similar between groups, except the HDS group had less coronary disease but was more sensitized (calculated panel reactive antibody ≥25%) compared to the LDS group. The most common immunosuppression regimen was the combination of tacrolimus, mycophenolate, and prednisone. Exposure to immunosuppression was similar between groups, except for a slightly higher dose of prednisone in the LDS group at months 3 and 6. Statin dose escalation occurred more in the LDS group. The HDS group had more evaluable intravascular ultrasound data.

**Figure.**
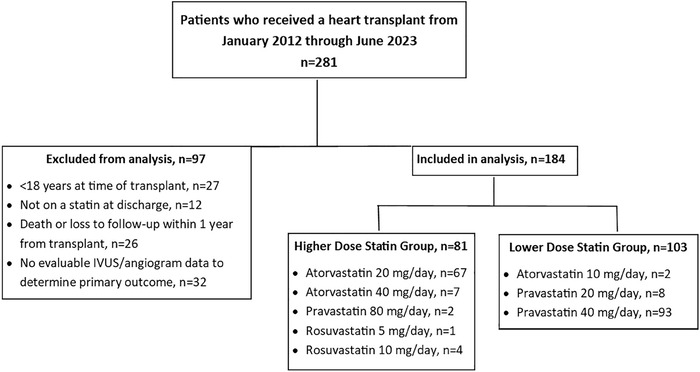
**Study population.** IVUS, intravascular ultrasound.

**Table 1.** Baseline, Perioperative, and Postoperative Patient and Clinical Characteristics by Treatment Group, n=184

Variable	Higher Dose Statin Group, n=81	Lower Dose Statin Group, n=103	*P* Value
Age, years, median [IQR]	49.8 [41.7, 59.3]	54.8 [39.5, 63.3]	0.335
Female	28 (34.6)	34 (33.0)	0.824
Race			0.277
Black	34 (42.0)	39 (37.9)	
White	40 (49.4)	60 (58.2)	
Other	7 (8.6)	4 (3.9)	
Coronary artery disease	35 (43.2)	59 (57.3)	0.058
Hypertension	60 (74.1)	72 (69.9)	0.532
Diabetes	32 (39.5)	43 (41.8)	0.759
Smoking history	39 (48.1)	55 (53.4)	0.479
Low density lipoprotein, mg/dL, median [IQR]	82.8 [60, 102]	87 [63, 111]	0.116
Serum creatinine, mg/dL, median [IQR]	1.0 [0.80, 1.30]	0.9 [0.80, 1.20]	0.102
Previous ventricular assist device	32 (39.5)	43 (41.8)	0.759
Heart-kidney transplant	5 (6.2)	2 (1.9)	0.243
Heart-liver transplant	2 (2.5)	0	0.192
Hepatitis C virus nucleic acid test–positive donor	2 (2.5)	4 (3.9)	0.696
Cytomegalovirus mismatch	34 (42.0)	45 (43.7)	0.816
Treated cytomegalovirus viremia	11 (13.6)	17 (16.5)	0.584
Calculated panel reactive antibody ≥25%	9 (11.1)	4 (3.9)	0.057
Anti-thymocyte globulin induction	33 (40.7)	33 (32.0)	0.222
Medications at discharge			
Aspirin	76 (93.8)	96 (93.2)	0.865
Tacrolimus	81 (100)	101 (98.1)	0.504
Cyclosporine	0	2 (1.9)	1
Mycophenolate	81 (100)	102 (99.0)	1
Azathioprine	0	1 (1.0)	1
Prednisone	81 (100)	102 (99.0)	1
mTOR inhibitor	0	2 (1.9)	1
mTOR inhibitor initiated within 1 year posttransplant	12 (14.8)	13 (12.6)	0.666
Tacrolimus level, ng/mL, mean ± SD			
3 months	11.3 ± 3.30	10.5 ± 3.34	0.065
6 months	9.6 ± 2.45	9.9 ± 3.58	0.582
12 months	8.5 ± 2.34	8.4 ± 3.32	0.805
Mycophenolate dose, mg, mean ± SD			
3 months	2,108 ± 1,010	2,060 ± 1,057	0.766
6 months	1,804 ± 1,083	1,841 ± 1,100	0.820
12 months	1,609 ± 1,121	1,510 ± 1,108	0.537
Prednisone dose, mg, mean ± SD			
3 months	7.5 ± 2.62	9.9 ± 5.78	0.001
6 months	3.6 ± 2.10	4.5 ± 3.07	0.027
12 months	2.2 ± 2.30	2.4 ± 2.93	0.654
Statin dose escalation	13 (16.0)	32 (31.1)	0.019
Statin dose de-escalation	6 (7.4)	3 (2.9)	0.185
Days on statin, median [IQR]	365 [365, 365]	365 [238, 365]	0.0544
Evaluable intravascular ultrasound data	74 (91.4)	77 (74.8)	0.004

Note: Data are presented as n (%) unless otherwise indicated.

IQR, interquartile range; mTOR, mammalian target of rapamycin.

The primary outcome, development of CAV, was not different between the HDS and LDS groups (32.1% vs 31.1%, respectively; *P*=0.881) (Table 2). The HDS group, however, had less biopsy-proven acute rejection (2.5% vs 12.6%, respectively; *P*=0.013) and less treated rejection (2.5% vs 17.5%, respectively; *P*=0.001) than the LDS group. Statin adverse effects were similar in both groups. A subgroup analysis of 151 patients with evaluable intravascular ultrasound data also showed no difference in CAV between the HDS and LDS groups (34.2% vs 28.0%, respectively; *P*=0.297).

**Table 2.** Patient Outcomes by Treatment Group, n=184

Outcome	Higher Dose Statin Group, n=81	Lower Dose Statin Group, n=103	*P* Value
Cardiac allograft vasculopathy	26 (32.1)	32 (31.1)	0.881
Biopsy-proven acute rejection	2 (2.5)	13 (12.6)	0.013
ISHLT cellular rejection ≥2R	2 (100)	9 (69.2)	
Antibody-mediated rejection grade ≥2	0	2 (15.4)	
Mixed	0	2 (15.4)	
Treated rejection^a^	2 (2.5)	18 (17.5)	0.001
Hemodynamically unstable rejection^b^	1 (1.2)	4 (3.9)	0.273
Adverse effects			
Liver dysfunction^c^	4 (4.9)	4 (3.9)	1
Myalgia^d^	13 (16.0)	23 (22.3)	0.286
Rhabdomyolysis^e^	1 (1.2)	0	0.440
**Subgroup with Evaluable Intravascular Ultrasound Data**			
**Outcome**	**Higher Dose Statin Group, n=76**	**Lower Dose Statin Group, n=75**	***P* Value**
Cardiac allograft vasculopathy	26 (34.2)	21 (28.0)	0.297

^a^Patients were considered treated for rejection if they received any of the following: augmented steroid therapy, anti-thymocyte globulin, rituximab, bortezomib, plasma exchange, or intravenous immunoglobulin.

^b^Hemodynamically unstable rejection was defined as treated rejection with an acute decrease in left ventricular ejection fraction (<45% or >25% from baseline) or the need for inotropes or mechanical circulatory support.

^c^Liver dysfunction was defined as aspartate transaminase or alanine transaminase >3 times the upper limit of normal.

^d^Myalgia was based on documentation of symptoms.

^e^Rhabdomyolysis was defined as creatinine phosphokinase >10 times the upper limit of normal.

Note: Data are presented as n (%).

ISHLT, International Society for Heart and Lung Transplantation.

Table 3 details the association between HDS treatment and CAV determined with logistic regression. Controlling for anti-thymocyte globulin induction, treated cytomegalovirus viremia, and calculated panel reactive antibody ≥25%, HDS was not associated with less CAV in the total population (adjusted odds ratio [OR] 1.10, 95% CI 0.58, 2.08; *P*=0.765) or in the subgroup with evaluable intravascular ultrasound data (adjusted OR 1.53, 95% CI 0.76, 3.11; *P*=0.237).

**Table 3.** Association Between Higher Dose Statin Treatment and Cardiac Allograft Vasculopathy

Outcome (Population)	Unadjusted Odds Ratio (95% CI)	*P* Value	Adjusted Odds Ratio (95% CI)	*P* Value
Cardiac allograft vasculopathy, total population (n=184)	1.05 (0.56, 1.96)	0.881	1.10 (0.58, 2.08)	0.765
Cardiac allograft vasculopathy, subgroup with evaluable intravascular ultrasound data (n=151)	1.44 (0.72, 2.89)	0.298	1.53 (0.76, 3.11)	0.237

Note: The logistic regression analysis was controlled for anti-thymocyte globulin induction, treated cytomegalovirus viremia, and calculated panel reactive antibody ≥25%.

## DISCUSSION

Statins are an integral part of the medication regimens after heart transplant, but whether higher statin exposure confers greater benefit is unclear. In this study, the occurrence of CAV was not different between the HDS and LDS groups, but less rejection occurred in the HDS group at 1 year.

Our finding that CAV was not attenuated with higher statin potencies mirrors the results from 2 observational studies.^7,8^ These studies, however, did not use intravascular ultrasound to evaluate CAV. We incorporated an intravascular ultrasound result in the definition of CAV for 2 reasons. First, rapid intimal thickening on intravascular ultrasound portends increased death and angiographic CAV at 5 years.^10^ Second, intravascular ultrasound can detect subtle changes in intimal thickening that angiography may not detect. Nevertheless, we found no difference in CAV per intravascular ultrasound or angiography between the statin intensity groups. Recently (2024), Huang et al conducted a study comparing pravastatin 20 mg/day and atorvastatin 20 mg/day and found no difference in the absolute maximal intimal thickness at 1 year.^18^

An explanation for the null findings across studies is that statin doses have not been high enough in the higher potency groups to further reduce CAV. Indeed, most patients categorized as receiving higher potency statins in these studies were on American College of Cardiology/American Heart Association (ACC/AHA) moderate-intensity statins.^19^ In our study, only 7 patients in the HDS group were on ACC/AHA high-intensity statins (atorvastatin 40 mg/day). Furthermore, a lack of difference in CAV seen between statin intensity groups may not have been detected because statins were often escalated in the LDS group within the first year after discharge, rendering differences in statin potencies minimal between groups. The collective data suggest that higher statin potencies do not further mitigate CAV in heart transplant recipients, but the effects on CAV outcomes of ACC/AHA high-intensity statins prescribed immediately after transplant must be further elucidated.

A seminal study by Kobashigawa et al demonstrated less rejection with hemodynamic compromise in heart transplant patients taking pravastatin compared to no statin.^20^ In our study, patients in the HDS group, despite being more sensitized, had less biopsy-proven acute rejection and treated rejection than patients in the LDS group. These findings, however, have not been replicated in similar studies comparing statin potencies in heart transplant patients.^8,18^ This discrepancy may be explained by our study population which was markedly different than the populations in the other studies referenced here. Compared to those studies,^8,18^ our population was slightly younger and had a higher number of both female and Black patients, which are all recognized risk factors for rejection.^16,21^ Our higher immunologic risk population may have derived greater immunomodulatory benefit from a higher statin potency because along with impairing natural killer cells, statins inhibit induction of major histocompatibility complex class II expression in a dose-dependent manner.^20,22^

Most episodes of rejection in this study were hemodynamically stable. The clinical significance of hemodynamically stable rejection remains a subject of controversy because patients with hemodynamically stable rejection require less urgent treatment and typically have better short-term prognosis compared to patients with hemodynamic compromise. The presence of biopsy-proven acute rejection in stable patients, however, has consequences. First, biopsy-proven acute rejection is a risk factor for the development of CAV.^23,24^ Second, biopsy-proven acute rejection is often treated with potent antirejection therapy, which is associated with infections and metabolic derangements from steroids.^25,26^ Indeed, prednisone doses were higher in the LDS group compared to the HDS group in this study. Taken together, the reduced rejection seen with higher statin exposure in the immunologically higher risk patients seen in our study vs other published reports^8,18^ is a unique finding that should be examined further in future investigations.

We found no increased adverse effects in the HDS group vs the LDS group, adding to the literature supporting the safety of higher statin potency in heart transplantation.^8,18,27^ Historically, safety concerns were raised about the use of higher statin potencies because of potentiation of statin drug exposure with cyclosporine, the first calcineurin inhibitor used in heart transplantation to prevent rejection.^28^ In the modern era, however, cyclosporine has largely been replaced by tacrolimus, which does not appear to augment statin exposure to the same degree.^29^ Heeney et al did not find increased hepatotoxicity or rhabdomyolysis when using ACC/AHA high-intensity statins in heart transplant recipients.^27^ In the Heeney et al study^27^ and ours, nearly all patients were on tacrolimus, supporting the notion that higher potency statins are safe in heart transplantation in the absence of cyclosporine.

In addition to the observational design, our study has limitations. First, we did not report decreases in low density lipoprotein for the 2 study groups, but the literature is conflicting on whether low density lipoprotein is correlated to CAV. Second, unaccounted changes in patient care may have occurred during the long study period and may have influenced outcomes. Third, adherence to statins could not be assessed. Fourth, a detection bias may have occurred in the HDS group given that more evaluable intravascular ultrasound data were available in the HDS group vs the LDS group. However, no difference was seen in the analysis of the subgroup with complete evaluable intravascular ultrasound data. Fifth, outcomes were examined only at the 1-year time point, and differences in CAV incidence between groups may be more apparent with longer follow-up.

## CONCLUSION

Our study suggests that an ACC/AHA moderate-intensity statin (eg, atorvastatin 20 mg/day) is a reasonable option after heart transplantation as its use appears to be safe and may reduce rejection. Attenuation of CAV, however, is likely not a benefit of moderate intensity statin therapy. Prospective studies should be considered to further elucidate the effects of higher statin potencies in heart transplant patients with high immunologic risk.

## Notes

*This article meets the Accreditation Council for Graduate Medical Education and the American Board of Medical Specialties Maintenance of Certification competencies for Patient Care and Medical Knowledge.*
